# Numerical analysis of the energy-storage performance of a PCM-based triplex-tube containment system equipped with arc-shaped fins

**DOI:** 10.3389/fchem.2022.1057196

**Published:** 2022-12-13

**Authors:** Azher M. Abed, Hosseinali Ramezani Mouziraji, Jafar Bakhshi, Anmar Dulaimi, Hayder I. Mohammed, Raed Khalid Ibrahem, Nidhal Ben Khedher, Wahiba Yaïci, Jasim M. Mahdi

**Affiliations:** ^1^ Air Conditioning and Refrigeration Techniques Engineering Department, Al-Mustaqbal University College, Babylon, Iraq; ^2^ Department of Mechanical Engineering, Tarbiat Modares University, Tehran, Iran; ^3^ College of Engineering, University of Warith Al-Anbiyaa, Karbala, Iraq; ^4^ Department of Physics, College of Education, University of Garmian, Kurdistan, Iraq; ^5^ Department of Medical Instrumentation Engineering Techniques, Al-Farahidi University, Baghdad, Iraq; ^6^ Department of Mechanical Engineering, College of Engineering, University of Ha’il, Ha’il, Saudi Arabia; ^7^ Laboratory of Thermal and Energetic Systems Studies (LESTE) at the National School of Engineering of Monastir, University of Monastir, Monastir, Tunisia; ^8^ CanmetENERGY Research Centre, Natural Resources Canada, Ottawa, ON, Canada; ^9^ Department of Energy Engineering, University of Baghdad, Baghdad, Iraq

**Keywords:** arc-shaped fins, latent heat storage, energy efficiency, phase change materials, heat transfer enhancement

## Abstract

This study numerically intends to evaluate the effects of arc-shaped fins on the melting capability of a triplex-tube confinement system filled with phase-change materials (PCMs). In contrast to situations with no fins, where PCM exhibits relatively poor heat response, in this study, the thermal performance is modified using novel arc-shaped fins with various circular angles and orientations compared with traditional rectangular fins. Several inline and staggered layouts are also assessed to maximize the fin’s efficacy. The effect of the nearby natural convection is further investigated by adding a fin to the bottom of the heat-storage domain. Additionally, the Reynolds number and temperature of the heat-transfer fluid (HTF) are evaluated. The outcomes showed that the arc-shaped fins could greatly enhance the PCMs’ melting rate and the associated heat-storage properties. The melting rate is 17% and 93.1% greater for the case fitted with an inline distribution of the fins with a circular angle of 90° and an upward direction, respectively, than the cases with uniform rectangular fins and no fins, which corresponded to the shorter melting time of 14.5% and 50.4%. For the case with arc-shaped fins with a 90° circular angle, the melting rate increases by 9% using a staggered distribution. Compared to the staggered fin distribution, adding an extra fin to the bottom of the domain indicates adverse effects. The charging time reduces by 5.8% and 9.2% when the Reynolds number (Re) rises from 500 to 1000 and 1500, respectively, while the heat-storage rate increases by 6.3% and 10.3%. When the fluid inlet temperature is 55°C or 50°C, compared with 45°C, the overall charging time increases by 98% and 47%, respectively.

## Introduction

Due to the environmental footprints left by the high consumption of fossil fuels and the quick depletion of conventional energy resources, the transition to renewable energy sources has become an inescapable strategy on a global scale (Kåberger, 2018; Mohammed, 2022). However, this transition potential is currently limited by the low thermal performance and inherent unpredictability of renewable energy systems (Rojas et al., 2018; Xu et al., 2022). Thermal energy storage (TES) systems are frequently advised (Langsdorf, 2011; Ghalambaz et al., 2021a) as a solution to this problem. Any energy system that allows for storing surplus heat during the day for later use during the night must include TES technologies. The effective primary way to increase energy efficiency and sustainability is the TES strategy, which has thus far caught the researcher’s attention (IRENA, 2020). The TES systems are utilized in energy conservation demands (Hasnain, 1998; Najim et al., 2022a), industry, commercial building (Heier et al., 2015), solar-powered energy systems (Fath, 1998; Shahsavar et al., 2020; Ansu et al., 2021), and thermal management of batteries (Mohammadi and Mohammadi, 2014; Keiner et al., 2019). In latent heat TES, the mechanism of thermal storage in the aforementioned applications is involved with phase-change materials (PCMs), which store thermal heat during the melting process as latent heat and then gives it off during solidification (Sardari et al., 2019; Zhou et al., 2022). The superiority of latent heat-storage mechanism using PCM is holding higher thermal capacity than sensible heat storage in addition to being melted/solidified at a relatively constant temperature (Sobolciak et al., 2015). Examples of phase-transition substances include various types of paraffin (Sobolciak et al., 2015; Ansu et al., 2020), salt hydrates (Wong-Pinto et al., 2020), fatty acids (Yuan et al., 2014), and esters (Sarı and Karaipekli, 2012) that are held in tanks, ponds, caverns, or underground aquifers depending on the type of the energy system involved. The ideal PCM possesses outstanding features during the phase change (melting/solidification) like high stability, chemical inertness, non-flammability, and high thermal conductivity to boost the thermal performance of the given energy system (Yamada, 2012). However, PCMs suffer from low thermal conductivity and diffusivity, which makes their usage limited. Thermal conductivity is a measure of heat transferred across a unit thickness of a material in a direction normal to the surface (Liao et al., 2018). In addition, thermal diffusivity is referred to as the thermal conductivity divided by the product of the density and heat capacity per unit mass at constant pressure (Burlatsky et al., 2009; Diakonova et al., 2020). Therefore, if these two parameters are weak in an energy system, the heat transfer rate is weak. Hence, numerous studies have been carried out to address this weakness by using nanoparticle additives (Kant et al., 2017; Abdulmunem et al., 2021), porous media (Zhou and Zhao, 2011; Talebizadeh Sardari et al., 2020), multi-stage PCM (Jin et al., 2017; Mahdi et al., 2020), fins (Ghalambaz et al., 2021b; Talebizadehsardari et al., 2021), *etc.*

In the context of the reliability and acceptability of a solar-powered energy system, Hassan et al. (2020) experimentally conducted a comparative study on a solar-powered shell and tube heat exchanger to propose a novel model with a higher thermal energy density. Their study involved three main configurations: no-fin tubes and longitudinal- and circular-finned tubes. According to the visual-based results, the PCM melting rate surprisingly decreased by 70% and 55% in the cases of circular and longitudinal fin configurations, respectively, compared with the no-fin case. Moreover, it was reported that using a circular fin configuration improves the thermal energy storage rate by 52%. Tiari et al. (2017) conducted an experimental study on a latent heat thermal energy system (LHTES) equipped with a heat pipe network. In their study, Rubitherm RT55 was considered the PCM, which was held inside a vertical cylindrical container, and heat pipes were distributed along the PCM container to improve the heat transfer rate. Another experimental study was carried out by Paria et al. (2016), where paraffin wax was employed as the storage medium within a horizontal shell, and a tube heat exchanger with annular fins clung to the tubes circulating the heat-transfer fluid (HTF). They also considered distilled water as the HTF with different flow rates to examine its effect on heat transfer performance. They found that higher fin density and HTF mass flow rate decrease the PCM melting time. Elmaazouzi et al. (2020) conducted a numerical study on the possible well-designed container for concentrated solar power plants using annular fin configurations. They showed that the PCM container’s optimum state positively improves the energy system’s overall thermal efficiency. They finally proposed an optimum configuration that causes a 50% enhancement in the heat transfer rate.

Among various enhancement technologies on PCM-based heat-storage systems, adding fins has been widely attended as a promising method in heat-transfer enhancement. Through an experimental and analytical study, Essa et al. (2020) investigated the effect of fin configurations on the thermal performance of an evacuated tube solar collector. In their study, helical fins were employed within paraffin in a container to make up for its low thermal conductivity and diffusivity. Their achievements were compared to a typical fin configuration. According to their results, a helical configuration of the fins was capable of obtaining, at most, a 1-h delay during the melting and solidification processes. Also, a higher peak temperature was achieved by the helical fin configuration compared with that of the conventional fin configuration. Nakhchi and Esfahani (2020) considered an LHTES equipped with lauric acid as the PCM equipped with novel stepped fin configurations. The PCM was heated from one side, where upward- and downward-stepped fin arrangements were taken into account to improve the natural convection heat transfer rate. They showed that a downward fin arrangement could successfully transfer the heat through the bottom of the container, where the heat gets stuck between the fins and the heated wall. This case showed improved melting time compared with the other stepped configurations of the fins. Some researchers (Tiji et al., 2020; Sun et al., 2021; Tiji et al., 2021; Wu et al., 2021; Najim et al., 2022b) have also investigated the effect of fin shape on the melting/solidification rate of PCM, such as incorporating a V-shaped fin configuration (Lohrasbi et al., 2016; Alizadeh et al., 2019). In this regard, Abdulateef et al. (2019) inserted triangular-shaped fins inside a PCM container in a triplex-tube heat exchanger to counteract the PCM’s low thermal conductivity. Furthermore, alumina nanoparticles were added to paraffin (RT82) as the PCM to improve the heat transfer rate further. Their study aimed to determine the optimum fin’s geometrical dimensions for a higher thermal energy-storage rate. Their results showed higher thermal density in the case with eight fins, each 141 mm in length, with a fin aspect ratio of 18%. It was also reported that the overall melting and solidification time prolongs in accordance with whatever increase in the aspect ratio and the fin length.

Although the literature review comprehensively covers the issue of low thermal conductivity enhancement of the PCM in different heat exchangers, fin configurations can impressively affect natural convection enhancement. They can also cause higher heat penetration inside the PCM domain by conduction, which requires further investigations in this field. Furthermore, there is a trade-off between the fin configuration inserted within an energy system and the overall volume of the materials involved. Hence, the optimum state of the fin’s volume is desired, while maximum thermal energy storage is achieved. This study considers a triple-tube heat exchanger integrated with Rt-35 as the PCM. In order to compensate for the low thermal conductivity and diffusivity of Rt-35, arc-shaped fin configurations are added to the PCM container to improve the conduction heat transfer area and the natural convection effect and cause higher heat penetration, and this part is considered the main contribution of this work. Furthermore, it is found that the inclination of fins and their angle of placement are influencing factors on the melting and energy-charging rate, which are investigated in this paper. Finally, an inline and staggered arrangement study is carried out to propose a well-designed case for the optimum cases.

## System description

**FIGURE 1:**
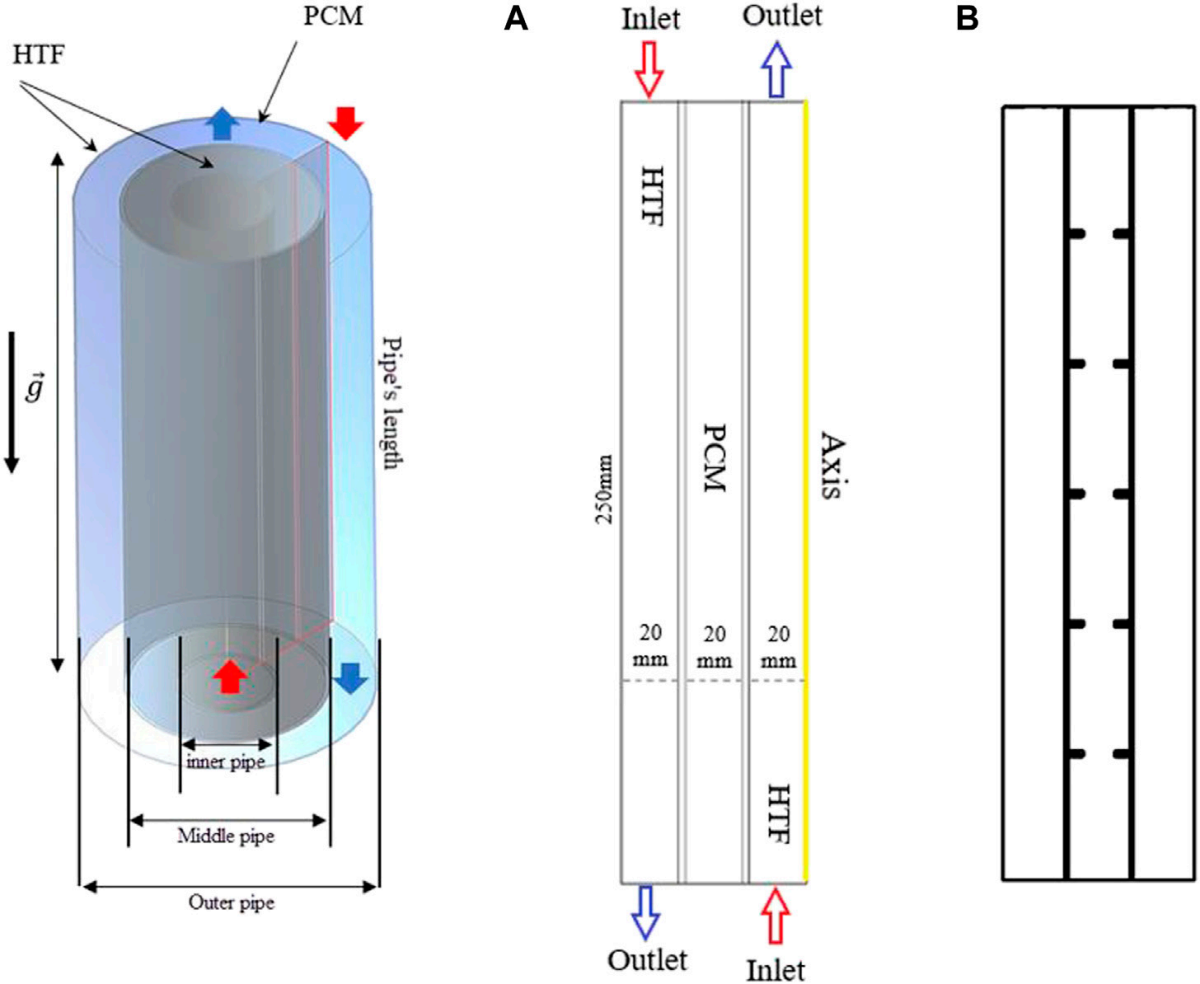
The schematic of a triplex tube heat exchanger with **(A)** no fins and **(B)** uniform fins.

A latent heat triplex-pipe heat exchanger with arc-shaped fins, as opposed to rectangular fins and a casing without fins, is the system under investigation. The PCM is situated in the central channel of the heat exchanger under examination, and water is passed through the inner and outer tubes as the working fluid. The tube length is 250 mm, and the inner, middle, and outer pipe sizes are 40, 80, and 120 mm, respectively. Figure 1 illustrates how the fluid flow and heat transfer are assessed under this study’s axisymmetric condition in both three-dimensional and two-dimensional axisymmetric structures. Due to the nature and system features under consideration, as well as the absence of a circumferential flow variation, the study was performed. The case without fins and the case with uniform rectangular fins are also displayed in Figure 1, called case 0 and case 1, respectively, in this study. The boundary conditions and dimensions of the system are also indicated for the system with no fins. Note that the dimensions of the unit are considered based on the literature (Al-Najjar et al., 2022; Chen et al., 2022).

**FIGURE 2:**
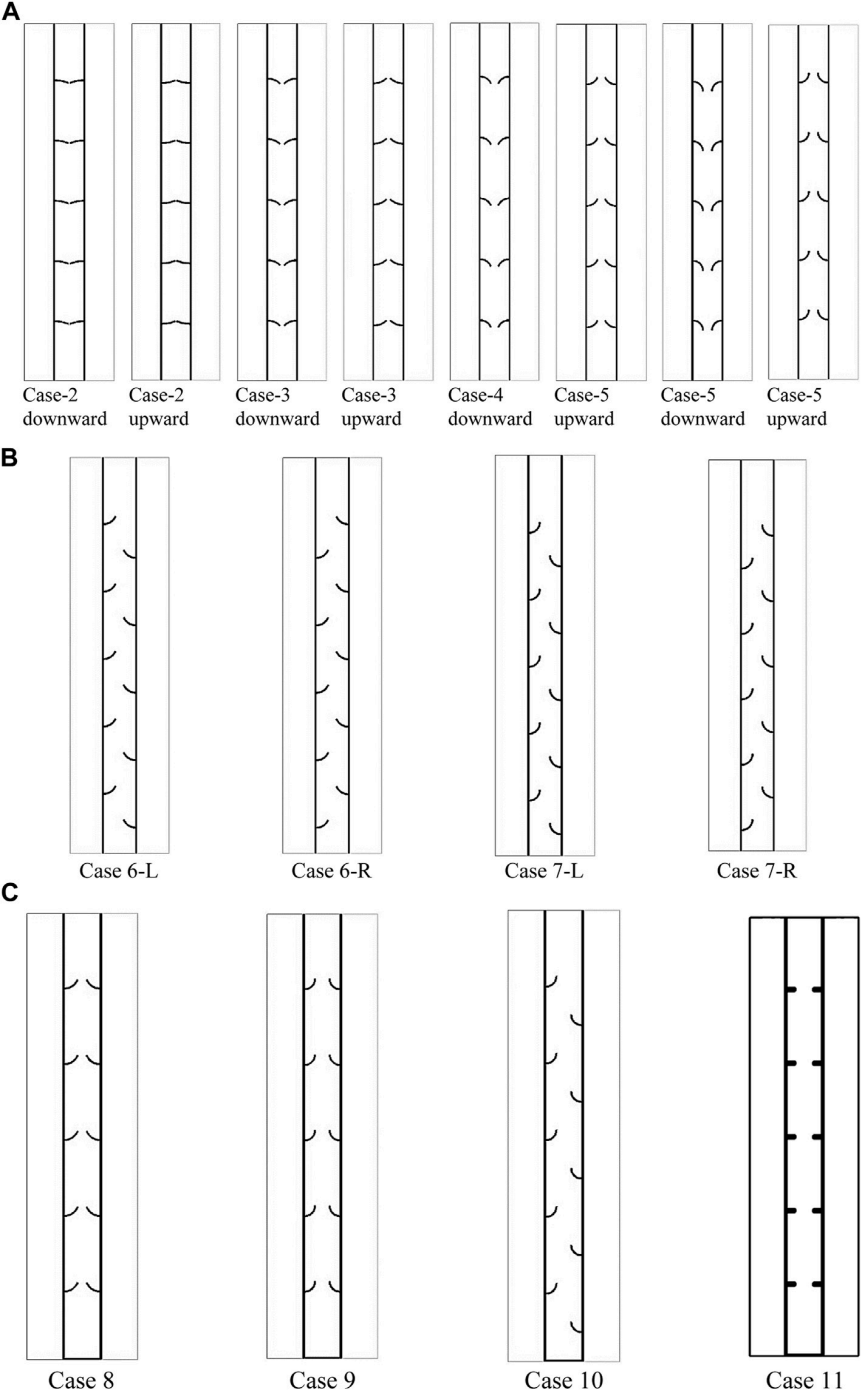
Schematic of the system with an arc-shaped fin **(A)** using different angles of 22.5, 45, 67.5, and 90° and different directions of downward and upward; **(B)** using different staggered arrangements for the fins; and **(C)** using an added fin to the bottom of the heat exchanger.

Four different shapes are studied for the arc-shaped fins in different directions, i.e., upward and downward. In all cases, the length of the fins is considered 10 mm with a thickness of 1 mm. Note that the dimension of the fins in the case of uniform rectangular fins is 5 × 2 mm. Different angles of 90, 67.5, 45, and 22.5° are proposed, as shown in Figure 2A.

After examining the angles and direction of the arc-shaped fins, the cases with arc angles of 67.5 and 90° are selected to examine the effect of the staggered arrangement of fins. Note that, as discussed later in the Results section, the upward direction of the fins shows a higher performance than the downward movement. Thus, for further analysis, the upward movement is considered. Figure 2B illustrates the schematic of the system, examining the effect of the staggered arrangement of the fins. Two different staggered formats, i.e., left-staggered and right-staggered, are discussed. In Figure 2B, case 6-R is case 4 in the upward direction, where the fins are in the staggered configuration from the right side of the heat exchanger (inner pipe). Case 6-L is case 4 in the upward direction, where the fins are in the staggered configuration from the left side of the heat exchanger (outer pipe). Cases 7-R and 7-L are also related to case 5 in the upward direction.

After studying the staggered configuration, it was found that the right staggered array results in a higher performance, which is discussed in the following sections. Thus, these systems (cases 6-R and 7-R) are compared with a case equipped with arc-shaped fins, uniform configuration, and an added fin to the bottom of the heat exchanger. The cases were also compared with the uniform rectangular-finned case with an added fin, as shown in Figure 2C. It is shown that due to modifying the natural convection effect at the bottom of the heat exchanger, it is adequate to add a fin at the bottom of the heat exchanger, as discussed by Tiari et al. (2017). In Figure 2C, case 8 and case 9 are related to the arc-shaped fins with the angle of 67.5° and 90° equipped with an added fin, respectively. Case 10 is also case 1 with an added fin to the bottom.

The best case is then compared with the no-fin case to show the amount of enhancement in the system’s performance. Paraffin RT-35 is used in the present study. The melting temperature of this PCM is suitable for HVAC applications. The properties of RT-35 are presented in Table 1.

**TABLE 1:** Thermodynamic properties of the PCM used in this study (Najim et al., 2022b).

Property	Value
$\rho_{l}$ [kg/m^3^]	770
$\rho_{s}$ [kg/m^3^]	860
$C_{p}$ [kJ/kgK]	170
Lf [kJ/kg]	2
K [W/mK]	0.2
μ [N.s/m^2^]	0.023
$T_{L}$ [ºC]	36
$T_{S}$ [ºC]	29
β [J/K]	0.0006

## Mathematical modeling

To represent the phase-change process of PCM, the enthalpy technique established by Brent et al. (Mahdi and Nsofor, 2017a; Talebizadeh Sardari et al., 2019) was applied. In this scheme, the liquid part was assumed to cover all the cells in the initial state of the computational field. To drive the governing differential equations, the following assumptions are used (Shahsavar et al., 2019; Shahsavar et al., 2020):

• Transient, laminar, and incompressible flow assumptions are applied to the flow of liquid PCM. However, the density variation caused by natural convection is solely considered in the buoyancy term of the momentum equation, $\rho =\rho_{ref}\left(1-\beta \left(T-T_{ref}\right)\right)$ (Zeneli et al., 2021).
• Gravity is considered positive in the downward *y*-direction.
• Adiabatic conditions are proposed at the exterior boundaries.
• No velocity slips exist at solid boundaries.

The continuity, momentum, and energy are then defined as follows (Wang et al., 2015):

$$\frac{\partial \rho }{\partial t}+\nabla .\rho \vec{V}=0$$ (1)

$$\rho \frac{\partial \vec{V}}{\partial t}+\rho \left(\vec{V}.\nabla \right)\vec{V}=-\nabla P+\mu \left(\nabla^{2}\vec{V}\right)-\rho \beta \left(T-T_{ref}\right)\vec{g}-\vec{S}$$ (2)

$$\frac{\rho C_{p}\partial T}{\partial t}+\nabla \left(\rho C_{p}\vec{V}T\right)=\nabla \left(k\nabla T\right)-S_{L}$$ (3)

The parameter ( $\vec{S})$ in Eq. 2 is included to calculate the effect of phase change on momentum, which is recognized as the velocity-inhibiting term in Darcy’s law (Esapour et al., 2016):

$$\vec{S}=A_{m}\frac{{\left(1-\lambda \right)}^{2}}{\lambda^{3}+0.001}\vec{V}$$ (4)

Here, the factor of the mushy area $A_{m}$ is set at 10^5^ depending on the literature (Ye et al., 2011; Mahdi and Nsofor, 2017b). To assess the phase-transition progression, λ (fluid part of PCM) is revealed as (Mat et al., 2013):

$$\lambda =\frac{∆H}{L_{f}}=\left\{\begin{array}{c} 0\;if\;T<T_{S}\\ 1\;if\;T>T_{L}\\ \frac{T-T_{S}}{T_{L}-T_{S}}\;if\;T_{S}<T<T_{L} \end{array}\right\}$$ (5)

The Boussinesq approximation is used in the model development so that the density variation due to temperature gradients is properly predicted (Mahdi and Nsofor, 2017c):

$$\rho =\rho_{ref}\left(1-\beta \left(T-T_{ref}\right)\right)$$ (6a)

The source term $S_{L}$ in the energy method is defined as

$$S_{L}=\frac{\rho \partial \lambda L_{f}}{\partial t}+\rho \nabla \left(\vec{V}\lambda L_{f}\right)$$ (6b)

The heat-storage rate during the melting process is measured as

$$\dot{E_{T}}=\frac{E_{end}-E_{ini}}{t_{m}}$$ (7)

where $t_{m}$ is the charging time and $E_{e}$ and $E_{i}$ are the whole PCM’s energy at the start and the end points of the melting process, respectively. E is the total heat, $MC_{p}dT$ is sensible case, and $ML_{f}$ is the latent case of the PCM.

### Initial and boundary conditions

The system is initially placed at a temperature of 15°C throughout the experiment ( $T_{ini}=50℃$ ). The HTF at constant velocity and temperature enters the unit from the inner and outer tubes at the inlet boundaries into the system. So, the boundary condition for the inlet can be described as follows:

$$at\;inlet,\;T_{HTF}=50℃\;and\;V_{HTF}=2.69\;\frac{cm}{s}$$ (8)

It should be noted that the aforementioned values are used as the base values for the comparison of different cases. For the assessment of HTF temperature and Reynolds number, the aforementioned values are changed for the inlet condition.

For the HTF at the outlet, the pressure outlet boundary condition is used, and the no-slip boundary condition is used for the walls. The boundary of the PCM domain is also considered adiabatic, except for the middle walls between the PCM tank and the HTF in the inner and outer tubes, which is a copper tube with a thickness of 2 mm used to transfer heat between the HTF and PCM.

## Numerical model

A grouping of the SIMPLE rule for pressure–velocity coupling and the Green–Gauss cell-based method was applied in ANSYS Fluent software to evaluate the thermal management and stream-governing formulations of PCM during the phase-change development. The QUICK variation procedure was utilized by the PRESTO method for the pressure correction formulations of the momentum and energy equations. After careful selection, the under-relaxation values are assumed as 0.3, 0.3, 0.5, and 1 for pressure correction, velocity components, liquid fraction, and energy equation, respectively. The convergence principles for terminating the iteration solution are 10-4, 10-4, and 10-6 for the continuity, momentum, and energy equations.

The mesh and the time step size independency examinations are carried out. Therefore, different grid sizes of 28,500, 43,000, and 81,620 are evaluated by applying a time step size of 0.2 s for the straight triplex unit. Table 2 introduces the charging period for different mesh densities. As demonstrated, the results are approximately the same for cell numbers 43,000 and 81,620; the mesh size of 43,000 is selected for the study’s next steps. Table 2 also lists the charging period for different time step sizes for the chosen cell number. As verified, the results are almost identical for the time step of 0.1, 0.2, and 0.4 s, mainly for the values 0.2 and 0.1 s. Therefore, 0.2 s is chosen for the time step size in this study.

**TABLE 2:** Effect of grid size and time step size on the charging time.

Number of cells	28500	43000			81620
Time step size (s)	0.2	0.1	0.2	0.4	0.2
Melting time	4644	4733	4727	4701	4739

**FIGURE 3:**
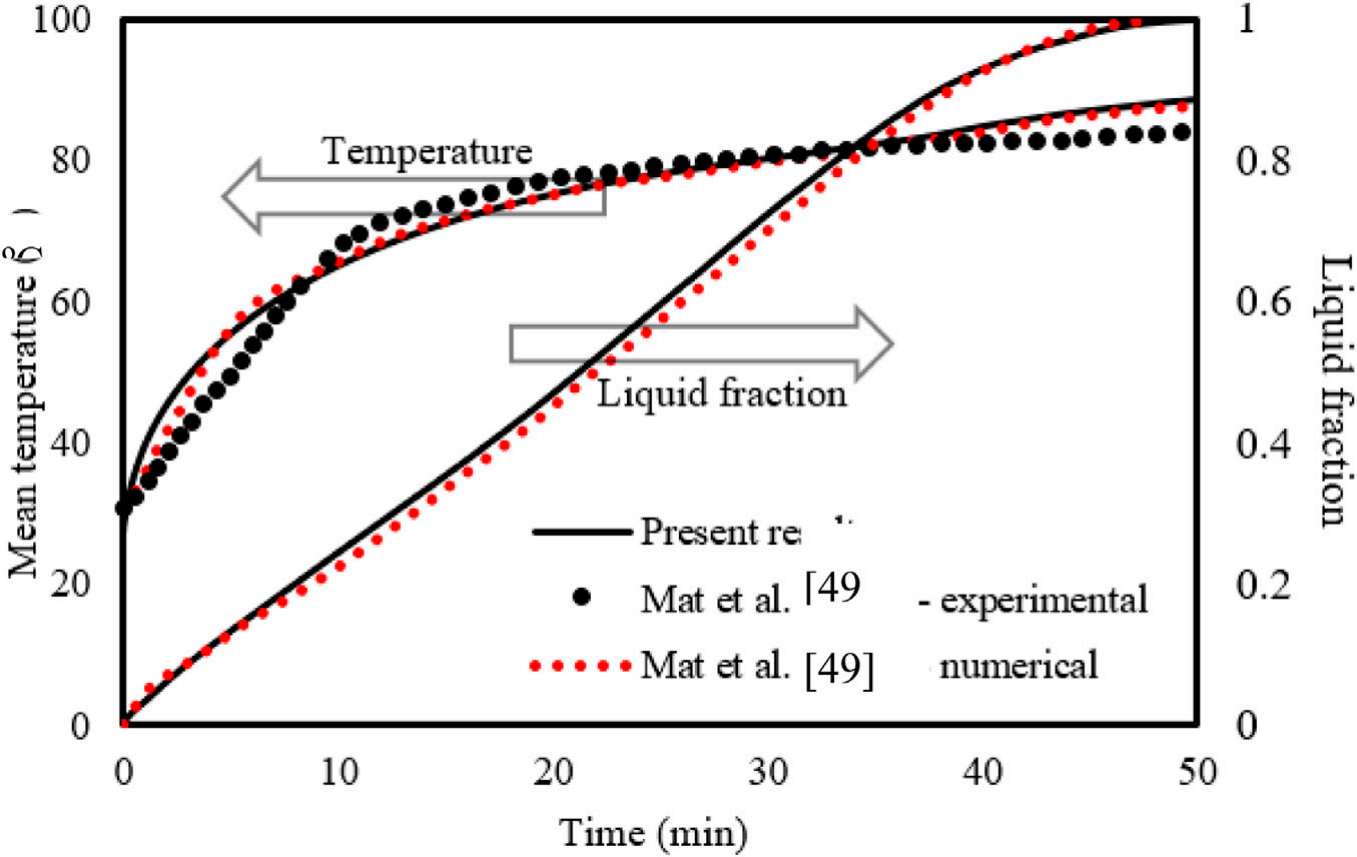
Evaluation of the current model’s temperature and liquid-fraction results compared to those in the study by Mat et al. (2013).

To investigate the appropriateness of the simulation progress, the outcomes of the study by Mat et al. (2013) were utilized as the benchmark, and the geometry applied in that achievement was rebuilt. Mat et al. (2013) numerically and practically assessed a double-tube heat exchanger system employing RT58 as the PCM. Mat et al.'s work was used as a referential work to validate the current model since the designs analyzed in the two studies are mostly the same. The referential work examined the attendance of integrated fins to the internal and external channels of the PCM shell in a staggered preparation, with the inner channel having a fixed wall temperature. A couple of effective parameters were applied to evaluate this model’s validity: the PCM’s thermal conduct and the growth of the liquid phase. Figure 3 illustrates the outcomes of the validation case, which confirms that the numerical and practical results of Mat et al. (2013) are close to the current model estimates for both the liquid fraction and average temperature of PCM.

## Results and discussion

In this study, first, the impact of the arc-shaped fins is placed in an upward direction (the direction of the arc is upward) and a downward direction (the direction of the arc is downward)). Four types of arches with downward and upward views are studied compared with the no-fin and uniform fin cases, as shown in Figure 2. The length of the fin is considered 10 mm with different angular sizes (90, 67.5, 45, and 22.5°) of the arc-shaped fins, as described previously. The thickness of the fins is 1 mm. The dimension of the fins in the case of the uniform fin is 2 × 5 mm. Then, the effect of the staggered arrangement of the fins is studied, followed by the effect of an added fin to the bottom of the heat exchanger. The final investigation includes the impact of temperature and velocity of the heat-transfer fluid (water), represented by the Reynolds number.

### Effect of arc-shaped fins with different angles (upward)

**FIGURE 4:**
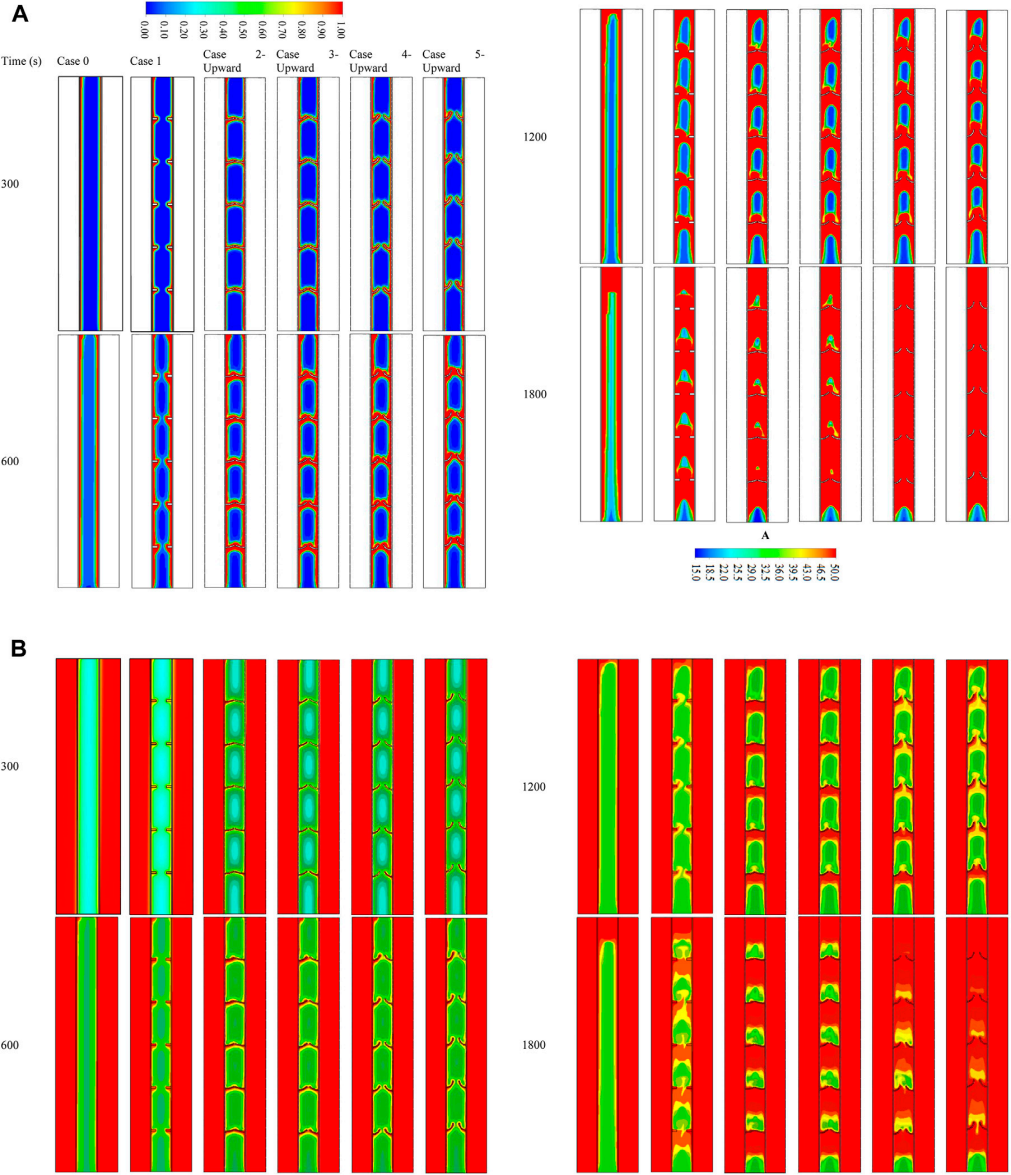
**(A)** Liquid fraction and **(B)** temperature development for various cases at different time steps (up to 1,800 s).

Integrating fins into the thermal energy storage system enhances the performance of the system due to expanding the thermal exchange surface area and growing the mean thermal conductivity of the system since the conductive value of the fin material is higher than that of the PCM. Furthermore, fins can carry heat from the walls deep to the PCM domain, causing faster phase change. The fins intensely influence free convection during the phase change since the fins create a barrier in the molten PCM movement. Four different upward view orientations of the arc-shaped fins at various time steps (up to 1800 s) were evaluated with the no-fin and uniformly distributed fin cases, and the liquid fraction contours are illustrated in Figure 4A. In the finless case, the PCM melts primarily in the areas beside the HTF walls. Progressively, more PCM melts, producing a wider molten layer attached to the wall and gathering at the upper side due to the natural convection. The PCM remains solid at the bottom zone of the unit. After 1800 s, only 68% of the PCM is melted. The liquid fraction percentage increases by utilizing fins in the system. The contours in the second column of the figure illustrate the uniform fin case. The spaces between the fins are 40 mm on each side (the inner and outer walls), with a 40 mm separation distance from the top and bottom sides. The liquid PCM performs beside the walls and over the fins, and the produced layer of the molten PCM expands steadily. A solid part is stuck above every couple of opposite fins, though the PCM phase between two opposite fins is changed. Within 1800 s, the total PCM melts were 89%, and the remaining solid part was split into six pieces. Because of the free convection, a movement of the liquid PCM creates a circulation mode, but the fins present a barrier to the movement bounding the free convection. In order to achieve the desired result, the shape and direction of the fins were changed and examined to improve the molten PCM’s progress.

The arc-shaped fins with an orientation of 22.5° (case 2—upward) are illustrated in the third column of Figure 4A. Because of the length of the fins and the slight slope, the PCM domain separates into several areas. Through the charging process of the PCM, the circulation achieves separately in those areas limiting the heat transfer in the domain. Increasing the orientation of the fins to 45° (case 3—upward) does not affect the heat transfer and the melting process. The gap between the fins is slightly open, which does not considerably affect the heat transfer. From the step times of the contours, apparent differences between the previous two cases regarding the melting time were not found. Opening the gates between the two opposite fins to 67.5° (case 3—upward) allows the PCM to circulate further in the domain, thus allowing more heat to transfer through and resulting in a faster melting process. Additionally, the fourth column shows that opening the gates (orientation of fins is 90° (case 4—upward)) allows more heat to transfer due to the natural convection, which also causes better circulation in the PCM domain.

Figure 4B is a counter map showing the temperature distribution throughout the melting duration of 1800 s for the cases discussed in the previous section. Because of the relatively short length of the system, the temperature of the HTF channels shows no significant changes throughout the melting mode. This is shown by red, the dominant color in the temperature distribution contours. In the case when there is no fin, the temperature rises in the area that is next to the walls of the HTF tube. The temperature of the PCM was predicted to have higher values in the areas closer to the solid wall. As a result of the convective heat transmission, the melted PCM accumulates at the top section, representing the highest temperature zone in the PCM domain. The temperature at the upper part reaches the equilibrium value with the HTF, and gradually, the equilibrium region expands through the domain. Within 1800 s, just 11% of the liquid PCM achieves the equilibrium state with the HTF temperature. The fin’s temperature in the uniformly distributed case almost arrives at the same temperature as the HTF because of the short length (5 mm) of the applied fins. As exposed, the fins deliver to the domain with a constant supply. Even though the small size of the PCM area has a similar temperature to the HTF temperature compared with the no-fin case, the average temperature is slightly higher in the case of inline fins. The orientation of the fins influences the thermal distribution of the PCM. The upward direction of the fins confines some PCM between the fins and the wall. In addition, using the curved fin with a dimension of 1 × 10 mm increases the heat transfer surface area and accelerates the melting process, in addition to enhancing the heat-storage rates. Due to the natural convection, the liquid PCM rises to the top of the domain, leaving the remaining solid part at the base. The fin’s dimensions and direction considerably influence the temperature of the PCM in the field. The low orientation angle of the fin causes a barrier to front the circulation of the molten PCM, resulting in lower heat exchange. Consequently, with a lower mean temperature registered with a higher slope, the gaps between the two opposite fins open, allowing the liquid PCM to flow through the entire domain, causing more heat exchange at the higher average temperature of the PCM. Cases with orientations of 67.5° almost have the same average temperatures because the fins create a shape allowing more PCM to circulate through the domain in both cases.

**FIGURE 5:**
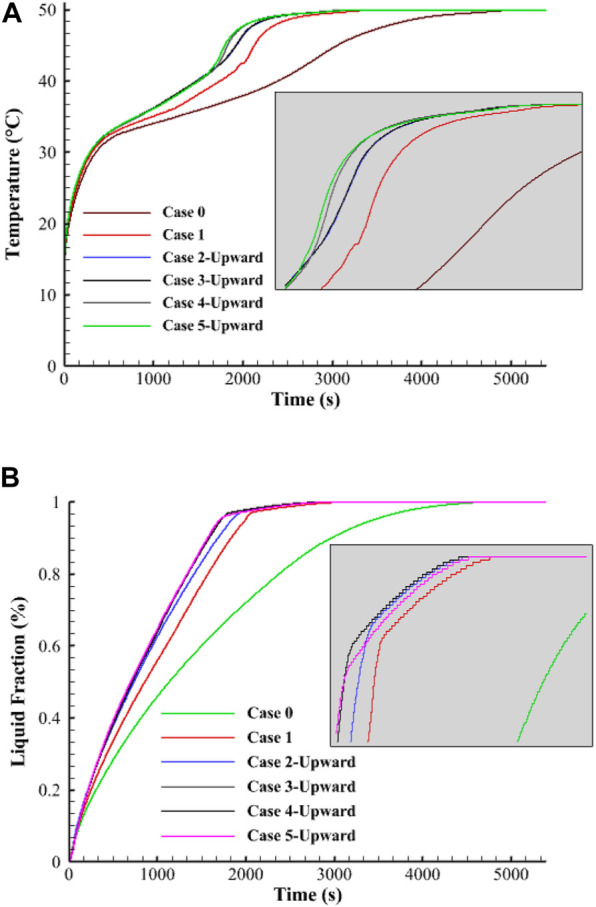
Variation of **(A)** temperature and **(B)** liquid fraction of the PCM for different studied cases during the melting process.

The average temperature sharply rises within the first 400 s due to the generation of the conduction heat transfer in the solid PCM, as shown in Figure 5A. The natural convection appears in the molten PCM attached to the walls and around the fins, causing a drop in the temperature rising rate. The cases of higher average temperature values are due to the arc-shaped fins having the largest surface area. The figure shows that the PCM temperature in the no-fin case has a softer increase with a lower rate. At the same time, the 90° orientation of the fin causes a higher increasing temperature rate and reaches the thermal equilibrium faster than in the other cases. Figure 5B illustrates the liquid fraction development for the mentioned cases till the fully melting process in the no-fin case. The phase change of the PCM performs smoothly till the entire melting, although the criterion for choosing the best case is reaching 95% of the liquid fraction. The melting time of 95% of the PCM in the no-fin case is 3435 s; however, this progress is much faster in the case of the 90° orientation arc-shaped fins with 1704 s. The use of fins causes a sharp melting rate due to the high heat exchange between the HTF and the PCM. By melting most of the PCM, the temperature difference between the two poles of the heat exchanger drops significantly, causing less heat to be delivered from the HTF to the PCM. Because of the long fix in the case of the 90° orientation fins, which provide a large surface area, and the direction of the fins, which allows softer circulation, this case is found to be the best case among the others.

Table 3 shows the storage heat rate and the melting time of the aforementioned cases. Note that in Table 3, the melting time and heat storage rates are presented for all the studied cases for the Reynolds number of 1,000 and inlet water temperature of 50°C. In the following sections, this table is referred to for studying the effect of various parameters. The storage heat rate registered for this case is 89.4 W, which is higher than the uniformly distributed fins, no-fins, 22.5°, 45°, and 67.5° orientation fins by 17%, 93%, 7%, 6.7%, and 1–1%, respectively. This procedure also applies the melting time found as 1704 s for the 90° fin direction case. This value is shorter than the aforementioned cases by 17%, 100.1%, 8%, 8.1%, and 0.8%, respectively.

**TABLE 3:** Heat storage rate and melting time for different studied cases during 95% of the melting process for Re = 1000 and Tin = 50°C.

Studied model	Heat storage rate (W)	Melting time (s)
Case 0	46.3	3435
Case 1	76.4	1994
Case 2—upward	83.6	1840
Case 3—upward	83.8	1834
Case 4—upward	88.4	1718
Case 5—upward	89.4	1704
Case 2—downward	80.4	1924
Case 3—downward	79.3	1953
Case 4—downward	82.2	1850
Case 5—downward	82.4	1846
Case 6-R	94.5	1567
Case 6-L	96.7	1529
Case 7-R	97.1	1526
Case 7-L	98.2	1508
Case 8	88.3	1845
Case 9	90	1810
Case 10	93.4	1708
Case 7-L	70.1	2406
Case 11	77.7	2078

### Effect of arc-shaped fins with different angles and downward direction

**FIGURE 6:**
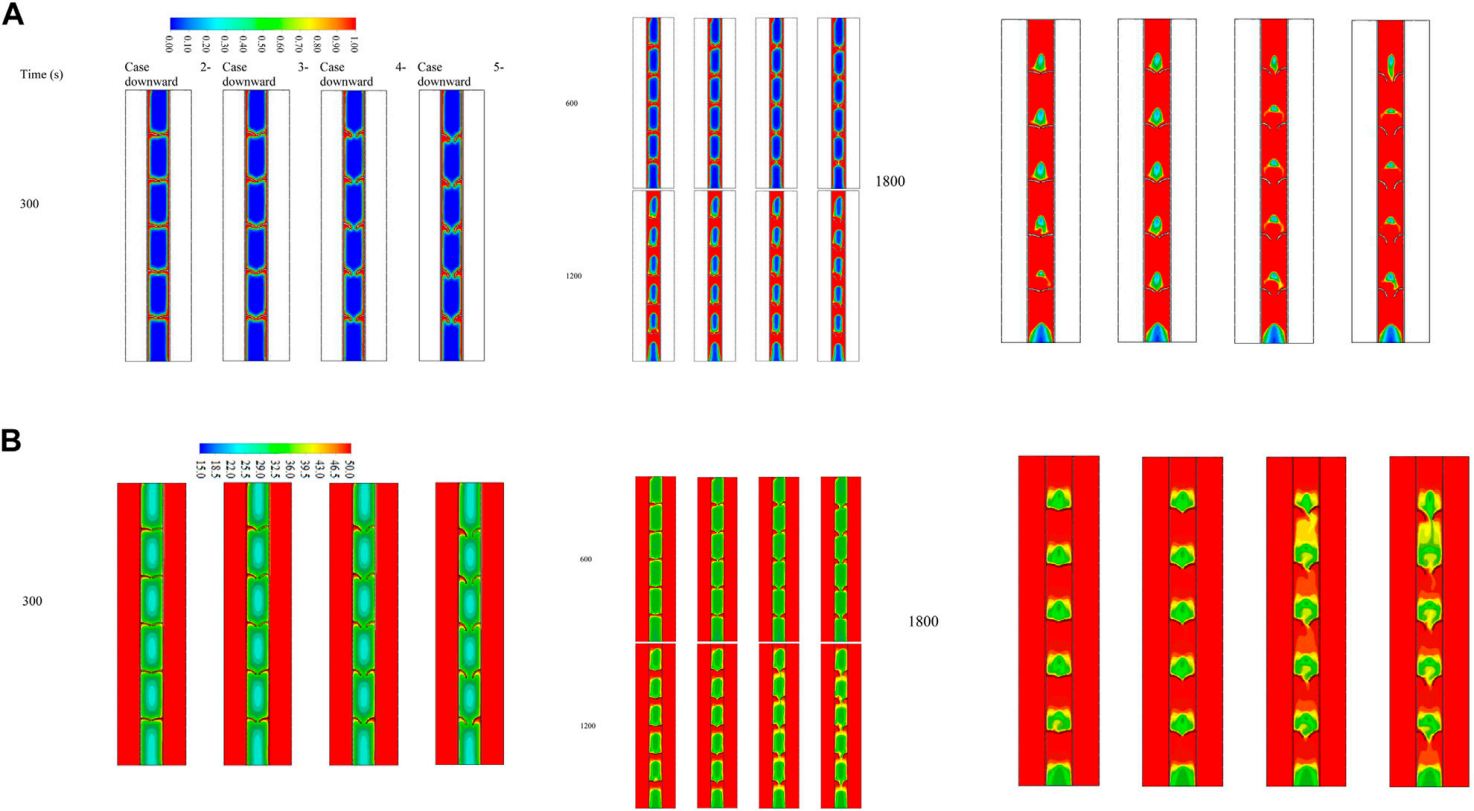
**(A)** Liquid fraction and **(B)** temperature distribution contour for various cases of the downward-view arrangement at different time steps (upto 1,800 s).

As the fin’s orientation greatly influences the melting process, the curved fins turned over to a great downward-view arrangement. Different directions have been considered, similar to the previous values, but the downward direction is chosen. Figure 6A shows the development of the liquid fraction for various cases during 1800 s. The same procedures are detected in the previous cases of 22.5° (case 2—downward) and 45° (case 3—downward) fin directions; however, the melting process passes slower steps for the cases of 67.5° (case 4—downward) and 90° (case 5—downward). The direction of the fins is the main reason for this behavior. In the downward-view fin, the inside of the curve is directed downward. This configuration helps the molten PCM to slip over the fin when it moves downward, but the liquid PCM is confined between the fins and the wall when it moves upward during the circulation. The outcomes of the cases of 67.5° and 90° are similar, and generally, the results have less improvement than those of the upward-view direction.

The temperature distribution contour for the downward-view configuration is shown in Figure 6B. The temperature increases beside the wall and around the fins and increases more by gaining more heat from the HTF. The temperature distribution behaves similar to that of the last cases since the liquid phase has a higher temperature and collects at the upper part of the domain and the confined zones between the fins, but the solid portion has a lower temperature relatively. The best case among the downward-view cases is the fin with an angle of 90°, and the average temperature at 1,800 s reaches 41°C.

Table 3 shows the melting time and the heat storage rate for a liquid fraction of 95% for the studied cases in the downward view. The best case found is the case of 90° orientation, while the heat storage rate is 82.4 W, which is higher by 2.5%, 93.9%, and 0.2% than the cases of 22.5°, 45°, and 67.5° angles of the fins. Similarly, the melting time for the best case (90°) is recorded at 1846 s, which is higher than the aforementioned cases by 78, 107, and 4 s, respectively.

### Effect of staggered arrangement for the two best cases (case 4—upward and case 5—upward)

**FIGURE 7:**
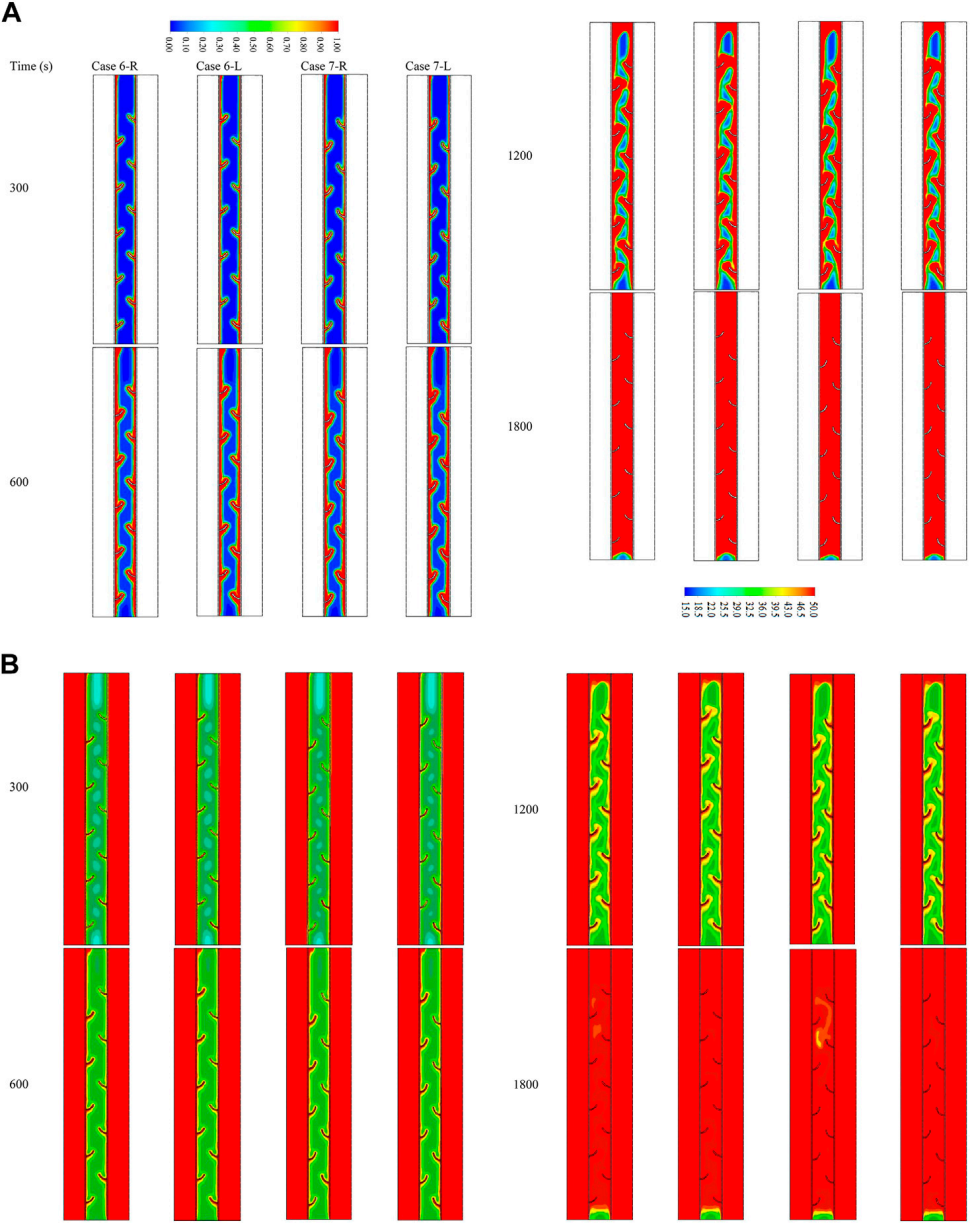
**(A)** Liquid fraction and **(B)** temperature distribution contour for various cases of the staggered fin at different time steps (up to 1,800 s).

Since cases 4—upward and 5–upward are found to be the best cases regarding the melting time and heat storage rate, these cases are selected for further investigation on the effect of the staggered layout of the fins. So, cases 6-R, 6-L, 7-R, and 7-L are explored in this section of the study. Figure 7A shows the liquid fraction development of the proposed cases within 1,800 s in different time steps. The melting process launches at the areas beside the walls and around the fins at the same rate for the cases due to the conduction effect. The liquid phase expands gradually to deeper zones of the PCM. The differences in the melting rate are found at 1,200 s of the process running, whereas the left staggered cases show an advantage over the right staggered case. The reason for this behavior is the more significant flow rate of the HTF through the left channel, causing higher heat exchange with the PCM. Within 1800 s, the whole PCM for the cases is melted, except the base part of the domain, which is still solid. Figure 7B shows the temperature distribution contours for the previous cases during 1,800 s of the operation. The temperature increases beside the walls and around the fins because of the heat exchange caused by the high-temperature difference between the PCM and the HTF. Since the liquid PCM gathers at the top of the domain, the temperature at that area reaches thermal equilibrium. Within 1800 s, most of the PCM in the left staggered reached thermal equilibrium, and the case of the 90° curved fin shows a higher average temperature (45°C) than the other cases.

The base area of the PCM shows a lower temperature as the PCM is still in a solid state. Table 3 shows the heat storage rate and the melting time through a period of 95% of the melting for the previous cases. Case 7-R is the best case among all the studied cases so far. The heat storage rate for this case is 98.2 W, which is higher by 3.9%, 1.6%, and 1.1% than that of cases 6-R, 6-L, and 7-L, respectively. Furthermore, the melting time for the best case is recorded at 1508 s, which is shorter by 59, 22, and 18 s than the aforementioned cases, respectively. The primary issue in the studied cases is the solid PCM remaining at the base of the domain. In order to achieve the desired result, a flat fin was inserted into the bottom of the field which helps in the melting process of the remaining solid part.

### Effect of added fin for the case with uniform fins compared with the best case with staggered arrangement

**FIGURE 8:**
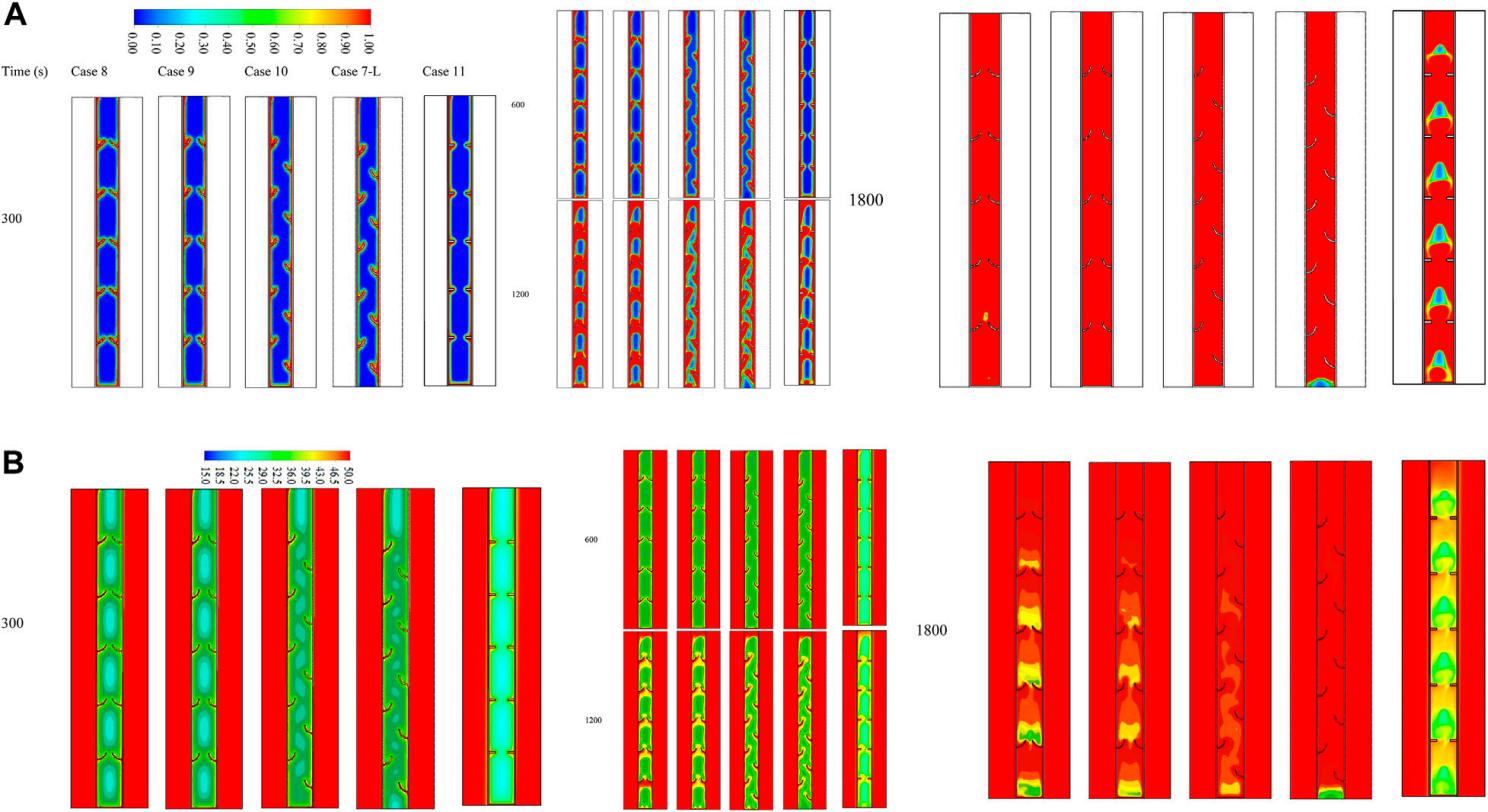
**(A)** Liquid fraction and **(B)** temperature distribution contours for various cases at different time steps (up to 1,800 s).

This subsection compares different cases of adding fin to the base of the domain, including cases 8, 9, 10, and 11 (rectangular fin with an added fin), compared with case 7-L, which is the best case in the previous section. Figure 8A shows the liquid fraction of the aforementioned cases at different time steps. Un-noticeable differences are shown earlier in the process (300 s); the only difference is that there is no melting PCM at the base of the domain without adding a flat fin. The liquid PCM expands gradually along the walls and around the fins. The effect of the added fins is clearly shown at 1200 s, as a portion of the solid PCM is still at the base of the case without adding a fin to the bottom (case 7-L); however, the PCM at the bottom of other cases is melting. The whole PCM is melted before 1800 s in both cases of 90° orientation fins with adding fin to the bottom of the domain. At 1,800 s, the solid PCM is clearly shown for both cases of regular uniform inline fins (line 5) and the case without adding a flat fin (line 4).

The temperature contours for the aforementioned cases show the same explained behaviors (Figure 8B). The temperature distribution pattern depends on the configurations and locations of the fins. Initially, the temperature rises along the wall and over the fins because of the high-temperature differences between PCM and the wall. The temperature of the HTF does not change because of the short length of the domain. At 1,800 s, the average temperature reaches a higher value for the cases of 90° sloped fins. The upper part of the domain matches the thermal balance with the HTF, and the average temperature drops as it goes downward.

Table 3 lists the heat storage rate and the charging period for the aforementioned cases during the fully charging process. Case 10 reveals a better performance than the other studied cases. The storage rate for this case is 93.4 W, which is higher by 5.8%, 3.8%, 20.2%, and 33.2% than cases 8, 9, 11, and 7-R, respectively. The melting time of the optimal case was recorded at 1,708 s, which is shorter than the aforementioned cases by 137, 102, 370, and 698 s, respectively. All the studied cases reveal that the 90° orientation fins, upward view with left staggered arrangement integrating with a flat fin at the base, is the best case regarding the charging period and the system’s general performance. Therefore, this case is further dependent on studying the effect of the inlet temperature and the flow rate presented by the Reynolds number.

### Effect of the Reynolds number on the best case

**FIGURE 9:**
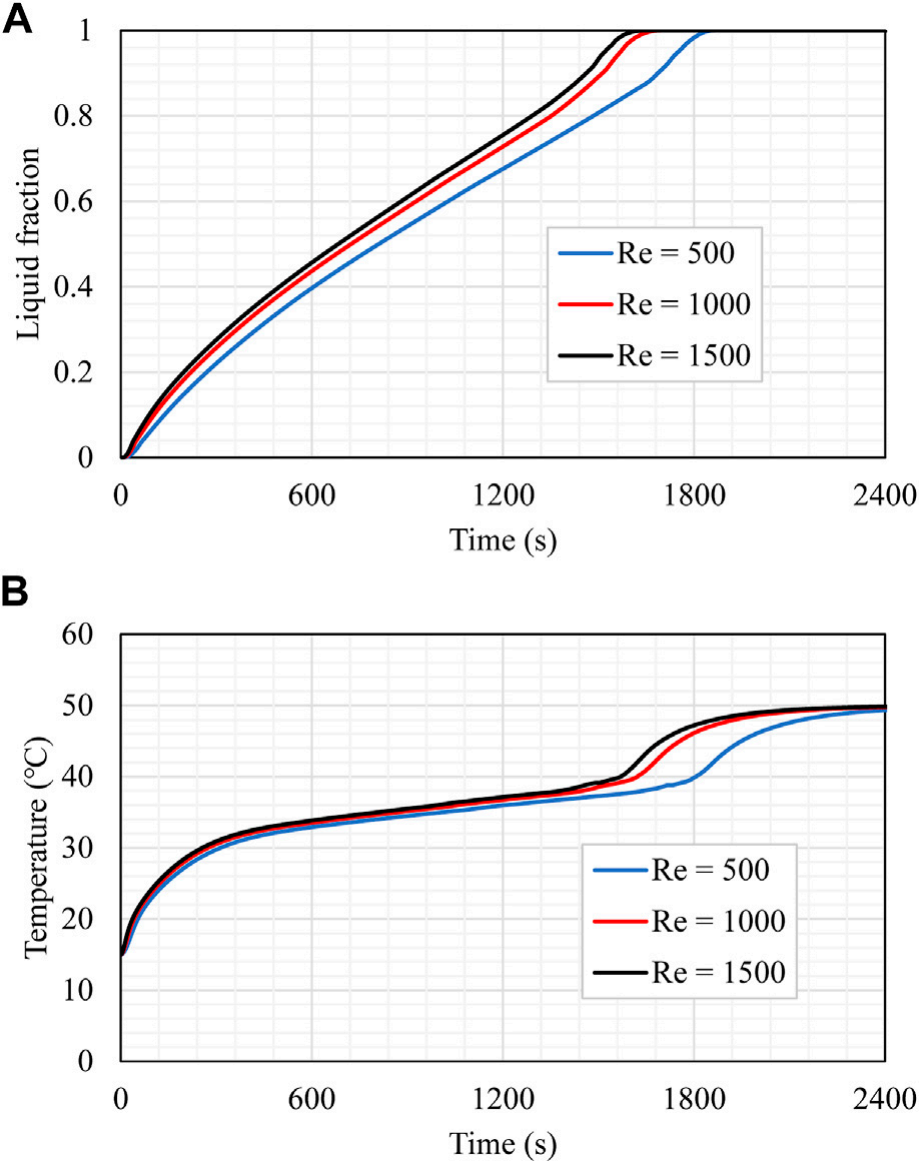
**(A)** Liquid fraction and **(B)** mean temperature rising of the PCM for different values of Re through the charging process.

The influence of the HTF flow rate (represented by Re) was explored for case 10 as it is the best-studied case. Various Reynolds numbers of 500, 1,000, and 1,500 (laminar flow range) were considered, as shown in Figure 9A. The velocity values related to the Reynolds numbers 500, 1,000, and 1,500 were 1.345, 2.69, and 4.035 cm/s, respectively. A higher velocity usually enhances the thermal ability and the convection heat transfer. The assumed Re was in the laminar flow range, and the impact of the HTF velocity on the charging process was studied. For all the values of the Re, the melting behavior and the slopes are almost similar. The period for the 95% melting of the PCM shows a clear advantage for the higher Re since the higher velocity provides a continuous high temperature of the HTF, resulting in a higher temperature difference between the HTF and the PCM and causing higher heat transfer. Figure 9B shows the average temperature development of the PCM for the aforementioned values of the Re. The PCM’s average temperature in the case of the higher Re shows higher values along the melting process, as the high Re provides a higher temperature difference between both operation materials. The temperature increases sharply at the beginning of the melting process due the thermal conduction, and then the gradient drops because of the appearance of the convection heat transfer, which is caused by the melting process. The variation in the slope, revealed for all Re, began at 1,600 s and was produced by generating liquid, which established a free convection impact. Although changes in the unit efficiency are slight when using various values of Re, minor enhancement concerning one parameter should be studied to enhance the entire system’s operation.

Table 4 reveals that the increase in Re from 500 to 1,000 and 1,500 drops the charging time by 5.75% and 9.15%, improving the heat storage rate by 6.18% and 10.2%, respectively. In summary, a greater Re value enhances the functioning of the unit by decreasing the charging period and raising the storage rate.

**TABLE 4:** Energy storage rate and melting period for case 10 for different values of Re during the charging process.

Studied model	Heat storage rate (W)	Melting time (s)
Re 500	84.4	1875
Re 1,000	93.4	1708
Re 1,500	96.5	1651

### Effect of inlet temperature of HTF

**FIGURE 10:**
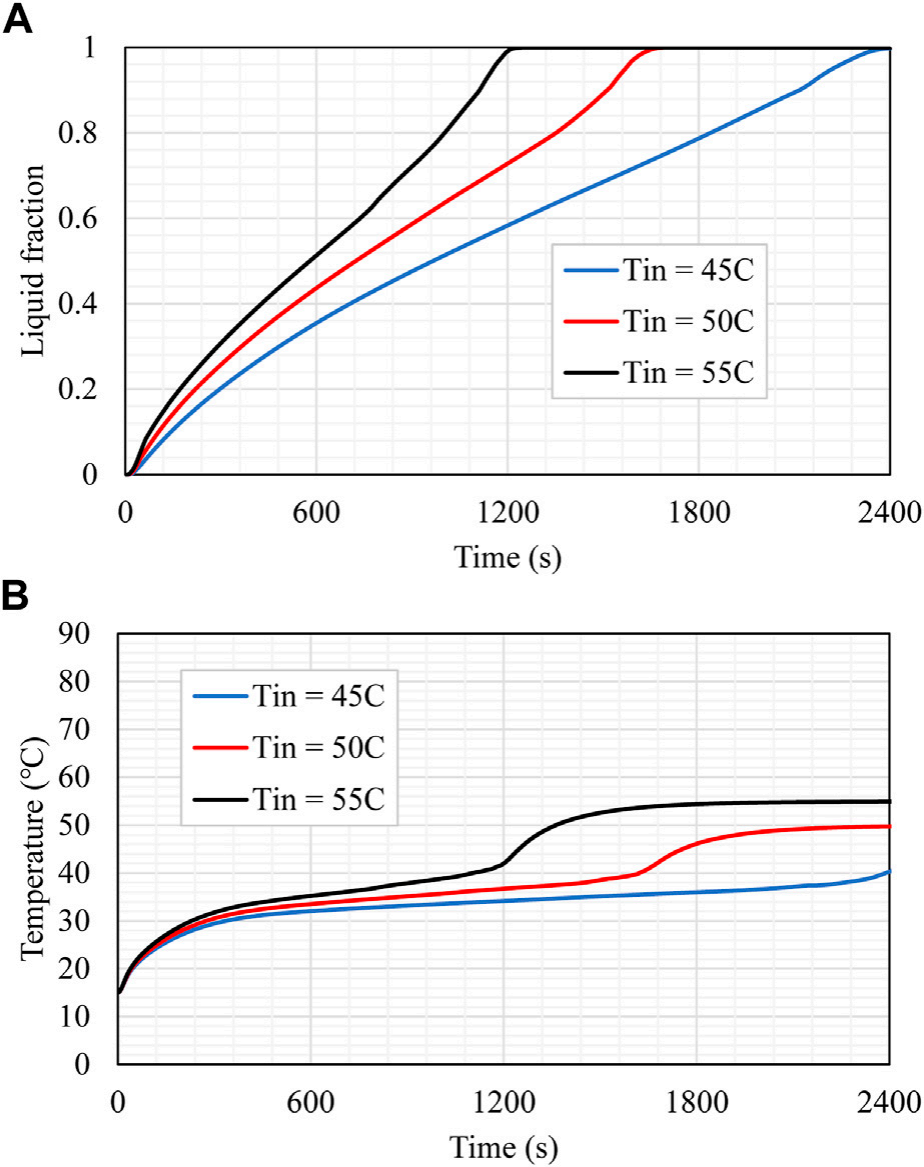
**(A)** Liquid fraction and **(B)** mean temperature rising of the PCM for different values of HTF inlet temperature during the melting process.

The HTF inlet temperature influences the charging process and is also investigated for the best-studied case (case 10). Three varying temperatures, i.e., 45, 50, and 55°C, are utilized for this assessment. According to Figure 10A, the difference in the temperature at the HTF inlet section significantly impacts the production of the molten PCM, and the figure shows that the charging time extends as the inlet temperature of HTF decreases. When the temperature at the inlet is greater, the temperature differential between the HTF and the PCM is also larger. This leads to an increased amount of heat being transferred from the HTF to the PCM, which expedites the charging process as a consequence. When the input temperature increases from 45 to 50°C or 55°C, the charging time is reduced by 350 s and 1,244 s, respectively. When employing the hotter HTF, the temperature of the PCM is more capable of attaining thermal equilibrium in a shorter amount of time. It took 1800 s for the PCM to achieve thermal equilibrium when the inlet temperature was 55°C. However, this period increased to 2,200 s and 2,400 s when the inlet temperature was 50°C or 45°C, respectively, as shown in Figure 10B. This outcome results from a high-temperature variance, which causes a greater heat transfer between the HTF and the PCM. As a result, the thermal equilibrium is reached more quickly in cases when the HTF temperature is at its maximum. The effects of the temperature of the incoming HTF are listed in Table 5. Compared to the charging duration at an inlet temperature of 45°C, which was 2,508 s, the total charging period rose by 98% when the inlet temperature was 55°C and by 47% when the inlet temperature was 50°C. When the inlet temperatures are 55 and 50°C, the heat storage rate was increased by 105% and 48%, respectively. The heat storage rate was 63 W when the inlet temperature was 45°C.

**TABLE 5:** Heat storage rate and melting time for case 10 for different values of HTF inlet temperature during the melting process.

Studied model	Heat storage rate (W)	Melting time (s)
Tin 45	63	2508
Tin 50	93.4	1708
Tin 55	129.2	1264

## Conclusion

The primary factor limiting the phase-change response of these materials in various applications is the limited heat conductivity of PCM. To satisfy the requirements of the associated applications, this fault necessitates an improvement in thermal performance. The melting process is improved by adding expanded surfaces or fins to thermal energy storage devices by raising the domain’s effective thermal conductivity. In this work, arc-shaped fins of various sizes and orientations were used to alter the PCM’s underwhelming thermal response compared to systems with no fins and systems with uniform fins during the charging process of a triple-tube thermal energy storage system. The curvature, placement, and orientation of the fins, as well as the initial HTF variables, such as the flow rate and temperature, were taken into account in several simulations run for the analyzed designs. The phases and temperature distributions in the domain, along with the charging time and heat storage rate for each case, were all considered in the improvement assessment. As a means of improving the natural convection effect in the blind area, the impact of adding a fin to the system’s base was also examined. The findings showed that the arc-shaped fins could considerably enhance PCM’s melting rate and associated heat storage properties. The melting rate is 17% and 93.1% greater for the case fitted with an inline distribution of the fins with a circular angle of 90° and an upward direction, respectively, than those of uniform rectangular fins and no fins. For the instance of arc-shaped fins with a 90° circular angle, the melting rate increases by 9% when using a staggered distribution. The system with 90° orientation fins, an upward view, left staggered integration, and a flat fin at the base is the best example of the melting time and the system’s overall performance, according to all the analyzed scenarios. The melting time is inversely related to the Reynolds number, and the storage rate is directly proportional. The charging time was lowered by 5.8% and 9.2% when the Reynolds number rose from 500 to 1,000 and 1,500, respectively, while the rate of heat storage increased by 6.2% and 10.3%. In conclusion, a higher Reynolds number improves the device’s performance by reducing charging time and boosting the storage rate. Regarding the HTF temperature, it was demonstrated that when the inlet temperatures were 55°C and 50°C, respectively, the whole charging period was extended by 98% and 47% compared to the melting time when the inlet temperature was 45°C, which is 2,508 s. When the inlet temperatures were 55°C and 50°C, respectively, the heat storage rate increased by 105% and 48% compared to the melting power at 45°C, which is 63 W.

The idea of employing fins with varying degrees and orientations of curvature to thermal energy storage systems has not been previously investigated. When these approaches are used, numerical results from this work show a promising improvement in storage operation. Additionally, additional thoughts on the issues of pressure drop and thermal management might be undertaken beforehand. In future, this work could be expanded to include further parameters such as porous medium, nanoparticles, using larger sizes of the system with different configurations, and performing such studies experimentally.

## Data Availability

The raw data supporting the conclusion of this article will be made available by the authors, without undue reservation.

## Nomenclature

$A_{m}$: mushy zone constant
$C_{p}$ **(Jkg^-1^K^-1^)**: specific heat
**E (J)**: heat storage
g **(ms^-2^)**: gravity
k **(Wm^-1^K^-1^)**: effective thermal conductivity
$L_{f}$ **(Jkg^-1^)**: latent heat of fusion
t **(s)**: time
T **(°C)**: temperature
$T_{f}$ **(°C)**: air temperature
$T_{s}$ **(°C)**: solid temperature
$T_{Liquidus}$ **(°C)**: liquidus temperature
$T_{Solidus}$ **(°C)**: solidus temperature
$\vec{V}$ **(m/s)**: velocity

### Greek symbols

λ: liquid fraction
ρ **(kgm^-3^)**: PCM density
∆H **(Jkg^-1^)**: PCM latent heat

### Abbreviation

TES: thermal energy storage
PCM: phase-change materials
LHS: latent heat storage
HTF: heat-transfer fluid
LHTES: latent heat thermal energy storage
TTHX: triple-tube heat exchanger
